# Overexpression of bHLH95, a basic helix–loop–helix transcription factor family member, impacts trichome formation via regulating gibberellin biosynthesis in tomato

**DOI:** 10.1093/jxb/eraa114

**Published:** 2020-03-05

**Authors:** Yao Chen, Dan Su, Jie Li, Shiyu Ying, Heng Deng, Xiaoqing He, Yunqi Zhu, Ying Li, Ya Chen, Julien Pirrello, Mondher Bouzayen, Yongsheng Liu, Mingchun Liu

**Affiliations:** 1 Key Laboratory of Bio-Resource and Eco-Environment of Ministry of Education, College of Life Sciences, Sichuan University, Chengdu, Sichuan, PR China; 2 Department of Metabolic Biology, John Innes Centre, Norwich Research Park, Norwich, UK; 3 GBF Laboratory, Université de Toulouse, INRA, Castanet-Tolosan, France; 4 University of Maryland, USA

**Keywords:** Gibberellin, gibberellin biosynthesis, tomato, transcription factor, transcriptional regulation, trichome

## Abstract

Trichomes are epidermal protuberances on aerial parts of plants known to play an important role in biotic and abiotic stresses. To date, our knowledge of the regulation of trichome formation in crop species is very limited. Through phenotyping of the *Solanum pennellii*×*S. lycopersicum* (cv. M82) introgression population, we identified the SlbHLH95 transcription factor as a negative regulator of trichome formation in tomato. In line with this negative role, *SlbHLH95* displayed a very low expression in stems where trichomes are present at high density. Overexpression of *SlbHLH95* resulted in a dramatically reduced trichome density in stems and a significant down-regulation of a set of trichome-related genes. In addition to the lower trichome density, overexpressing lines also showed pleiotropic alterations affecting both vegetative and reproductive development. While most of these phenotypes were reminiscent of gibberellin (GA)-deficient phenotypes, expression studies showed that two GA biosynthesis genes, *SlGA20ox2* and *SlKS5*, are significantly down-regulated in *SlbHLH95-OE* plants. Moreover, in line with a decrease in active GA content, the glabrous and dwarf phenotypes were rescued by exogenous GA treatment. In addition, yeast one-hybrid and transactivation assays revealed that SlbHLH95 represses the expression of *SlGA20ox2* and *SlKS5* via direct binding to their promoters. Taken together, our study established a link between *SlbHLH95*, GA, and trichome formation, and uncovered the role of this gene in modulating GA biosynthesis in tomato.

## Teaser

This work provides new insight into the regulation of trichome formation by gibberellin which has not previously been described in tomato.

## Introduction

Plant trichomes are specialized epidermal cell structures present on aerial parts of plants including leaves, stems, and peduncles. Trichomes play important roles in plant physiology and development by providing physical or chemical barriers that protect plants against insect predation, UV radiation, and excessive transpiration (Ishida *et al.*, 2008). Depending on the species, trichomes are classified as unicellular or multicellular, glandular or non-glandular, and branched or unbranched (Werker, 2000; Pattanaik *et al.*, 2014).

Arabidopsis trichomes are typically unicellular, branched, and non-glandular. The initiation and regulation mechanisms underlying trichome formation have been thoroughly studied in Arabidopsis, and genetic and molecular analyses demonstrated that a complex network of transcription factors (TFs) is involved in this process. The R2R3 MYB, basic helix–loop–helix (bHLH), and WD40 repeat (WDR) proteins, form a trimeric MYB–bHLH–WDR complex that promotes the formation and development of trichomes by activating the expression of downstream target genes (Rerie *et al.*, 1994; Johnson *et al.*, 2002; Ishida *et al.*, 2007). Other TFs including TRANSPARENT TESTA 8 (TT8) and AtMYC1, NAC, and NTM1-LIKE 8 (NTL8) proteins also play pivotal roles in trichome development (Maes *et al.*, 2008; Symonds *et al*., 2011; Zhao *et al.*, 2012; Tian *et al*., 2017). In addition, phytohormones, especially gibberellins (GAs), jasmonates (JAs), and cytokinins (CKs), regulate trichome formation by modulating the expression of key regulatory genes in Arabidopsis (Schellmann and Hulskamp, 2005; Yang and Ye, 2013; Qi *et al.*, 2014). Although the regulatory network of unicellular trichome formation in Arabidopsis has been extensively studied, our knowledge of the initiation and development of trichomes in crop species remains scarce.

Considering the wide diversity of trichome types in tomato (*Solanum lycopersicum*), this species can potentially serve as a model system for studying multicellular and glandular trichomes, due to the extensive genetic resources available, the high-quality genome sequence, and other genomics resources along with a handful of reported trichome mutants among which are *chi1*, *coi1*, *hairless*, *odorless-2*, and *inquieta* (Li *et al.*, 2004; Kang *et al.*, 2010*a*, *b*, 2014; Jeong *et al.*, 2017). Tomato produces eight types of trichomes which can be divided into glandular (types I, IV, VI, and VII) and non-glandular (types II, III, V, and VIII) whose characterization is giving rise to an increasing number of studies (Luckwill, 1943; Simmons and Gurr, 2005; Glas *et al.*, 2012; Xu *et al.*, 2018). The HD-Zip protein Woolly was reported to physically interact with a B-type cyclin protein SlCycB2 to regulate type I trichome formation (Yang *et al.*, 2011). Moreover, the C2H2 zinc finger protein Hair (H), interacting with Woolly, was recently identified as a key regulator of type I trichome formation in tomato (Chang *et al.*, 2018). SlMX1, a MIXTA-like MYB TF, was shown to be involved in the formation of both glandular and non-glandular trichomes in tomato (Ewas *et al.*, 2016, 2017). In addition, down-regulation of *SlIAA15* or *SlARF3*, two auxin-related TF genes, led to reduced trichome density in tomato (Deng *et al.*, 2012; Zhang *et al.*, 2015). More recently, it was shown that down-regulation of *SlMYC1*, a bHLH TF gene, resulted in the production of smaller type VI glandular trichomes at lower densities, and the complete knockout of this gene led to total absence of type VI trichomes (Xu *et al.*, 2018), indicating that SlMYC1 is instrumental in type VI glandular trichome development in tomato. In addition, it was reported that JA is required for the formation and development of type VI glandular trichomes in tomato (Li, 2004; Yan *et al.*, 2013; Bosch *et al.*, 2014). However, the role of other hormones such as GAs in tomato trichome formation remains unclear. In spite of the progress made in uncovering some of the factors controlling trichome development in tomato, our understanding of the regulatory mechanisms underlying the development of different types of tomato trichomes remains largely incomplete.

Here, we report on a new bHLH TF, SlbHLH95, acting as a putative regulator of trichome formation identified by screening the *S. pennellii*×*S. lycopersicum* introgression population for altered trichome phenotypes. Overexpression of *SlbHLH95* in tomato resulted in reduced trichome density in stem, leaf, and young fruit. Moreover, *SlbHLH95*-overexpressing (OE) lines exhibited GA-deficient phenotypes that were rescued by exogenous GA application. The data provide new insight into the role of bHLH TFs in controlling trichome formation in tomato through the regulation of GA biosynthesis.

## Materials and methods

### Plant materials and growth conditions

Tomato (*S. lycopersicum*, Micro-Tom) plants were grown under standard greenhouse conditions set as follows: 14 h day/10 h night cycle, 25 °C/20 °C day/night temperature, 80% relative humidity, and 250 μmol m^−2^ s^−1^ light intensity.

### Plant transformation

*Agrobacterium tumefaciens*-mediated tomato plant transformation was performed as described in Wang *et al.* (2005). The transformed lines were then selected on a kanamycin-containing medium. Homozygous lines from the F_3_ or later generations were used in all experiments.

### Subcellular localization of SlbHLH95 protein

To investigate the subcellular localization of the SlbHLH95 protein, the coding sequence of *SlbHLH95* without the stop codon was amplified by PCR and then cloned into pART2a to generate the 35S::SlbHLH95-GFP (green fluorescent protein) fusion gene. The vector bearing the fusion construct SlbHLH95–GFP and the control GFP vector were individually transformed into leaves of *Nicotiana benthamiana* using a 2 ml needleless syringe. The transformed samples were incubated in the dark at 22 °C for 48 h, then the subcellular localization of each expressed protein was visualized using a confocal microscope (Leica, TCS SP5 II). Images of transformed tobacco cells were captured using a ×40 objective lens, using bright field, GFP (excitation/emission: 488/498–548 nm), and DAPI (excitation/emission: 405/421–523 nm) filters.

### RNA extraction and RT–qPCR

Total RNAs from different tissues were isolated using a Plant RNA Purification Reagent (Invitrogen, cat. no. 12322-012) according to the manufacturer’s instructions. cDNA was synthesized from 1 μg of total RNA using a PrimeScript™ RT reagent Kit with gDNA Eraser (Takara Bio, Kusatsu, Japan, AK4201). cDNA products were diluted to 2.5 ng μl^–1^ and used as templates for the qPCR. Quantitative reverse tanscription–PCR (RT–qPCR) was performed using the Bio-Rad CFX384 device. Each reaction (10 μl) consisted of 5 μl of iTaq™ Universal SYBR Green Supermix (Bio-Rad, Hercules, USA, #172-5124), 1 μl each of forward and reverse primers, and 3 μl of cDNA. Thirty-nine amplification cycles were performed (pre-incubation at 95 °C for 2 min followed by each cycle consisting of 5 s at 95 °C, 10 s at 60 °C, and added melting curve analysis during 65–95 °C). The results were calculated using Bio-Rad CFX Manager software. The relative expression of each gene was calculated by the ∆∆Ct method (Schefe *et al.*, 2006) using *SlActin* as internal control. The primer pairs for RT–qPCR were designed using Primer3Plus (http://www.primer3plus.com) and blasted at the NCBI database to check their specificity (the primers used for qRT–PCR are listed in Supplementary Table S3 at *JXB* online).

### Fruit shape analysis

Full size maturing (7 d post-breaker stage) freshly harvested fruits were cut with a new razor blade longitudinally, scanned at 300 dpi, and analyzed using Tomato Analyzer v3.0 (Rodríguez *et al.*, 2010). Then fruit shape index and fruit shape triangle, two parameters characterizing the shape of the fruit, were obtained after analysis. Fruit shape index is the ratio of the maximum height length to the maximum width of the fruit, and fruit shape triangle is the ratio of the proximal end width to the distal end width (Brewer *et al.*, 2007).

### SEM analysis

Fresh tomato leaves or stems (three biological replicates) collected from wild-type (WT) and transgenic plants were fixed for 24 h in a solution of 2.5% paraformaldehyde, 2.5% glutaraldehyde buffered with 0.1 M sodium cacodylate, pH 7.4 (Electron Microscopy Sciences, Hatfield, PA, USA). Samples were dehydrated in the following ethanol concentrations (30, 50, 70, 80, 90, 100, and 100%), each treatment lasting 10 min. Samples were then critical point dried with liquid CO_2_ (Quarum K850 CPD), mounted, and sputter coated with a 5 nm thin layer of platinum (LEICA EM MED 020). Images were acquired with a scanning electron microscope (SEM Quanta 250 FEG FEI) at 5 kV, spot size 3 or 2 with a working distance of 1 cm.

### RNA-Seq analysis and data processing

Global expression of tomato genes was determined by replicated strand-specific Illumina RNA sequencing (RNA-Seq). Paired-end RNA-Seq was carried out using Hiseq 2500 (150 bp paired ends) by Novogene (China). WT and *SlbHLH95-OE83* RNAs were extracted from 4-week-old leaves with three biological replicates. Prior to sequencing, the quality of purified RNA was checked with the Agilent2100 Bioanalyzer (RIN ≥6.3).

Raw data (raw reads) of fastq format were firstly processed through in-house perl scripts. In this step, clean data (clean reads) were obtained by removing reads containing adaptor, reads containing poly-N (>10% unknown base per read), and low-quality reads (>50% of Q_phred_ ≤20 bases per read) from raw data. At the same time, Q20 (probability of incorrect base call, 1 in 100), Q30 (probability of incorrect base call, 1 in 1000), and GC content of the clean data were calculated. All the downstream analyses were based on the clean data with high quality. Trimmed reads were then mapped to the *S. lycopersicum* reference genome and gene annotation ITAG2.4 (Tomato Genome Consortium, 2012) using TopHat-2.0.12 (Trapnell *et al.*, 2009) calling Bowtie 2.2.3 (Langmead and Salzberg, 2012).

Differential expression analysis was performed using the DESeq R package (1.18.0) (Anders and Huber, 2010). DESeq provides statistical routines for determining differential expression in digital gene expression data using a model based on the negative binomial distribution. The resulting *P*-values were adjusted using the Benjamini–Hochberg’s approach (Benjamini and Hochberg, 1995) for controlling the false discovery rate. Genes with an adjusted *P*-value < 0.05 found by DESeq were assigned as differentially expressed genes (DEGs).

Gene Ontology (GO) enrichment analysis of DEGs was implemented by the GOseq R package, in which gene length bias was corrected. GO terms with a corrected *P*-value <0.05 were considered as significantly enriched within DEGs (Young *et al.*, 2010). Then, we carried out the statistical enrichment of the differential expression of DEGs in Kyoto Encyclopedia of Genes and Genomes (KEGG) pathways using KOBAS software (Mao *et al.*, 2005; Kanehisa *et al.*, 2008).

All raw-sequence reads data were uploaded in the NCBI Sequence Read Archive (SRA; http://www.ncbi.nlm.nih.gov/Traces/sra) with accession number SRP144705.

### GA content analysis

GA measurements were conducted by Wuhan Metware Biotechnology Co., Ltd. (Wuhan, China). Leaf material was collected from WT and *SlbHLH95-OE* plants at the 4-week-old stage. Three replicates were performed for each genotype. Frozen plant materials were ground using a mixer mill (MM 400, Retsch, Germany) for 1 min at 30 Hz. Then, 200 mg of the powder sample was extracted overnight at 4 °C with 1500 μl of 70% (v/v) acetonitrile, and ultrasound-assisted extraction was carried out for 30 min at room temperature with vortexing (15 s) and centrifugation (14 000 rpm for 10 min). From each sample, 1000 μl of supernatant was collected, then evaporated to dryness under a nitrogen gas stream at room temperature, reconstituted in 100 μl of 80% (v/v) methanol, and diluted to 800 μl with water. The extracts were passed through the SPE cartridge (200 mg, 3 ml; CNW) and evaporated to dryness under a nitrogen gas stream at room temperature (Ma *et al.*, 2008; Hao *et al.*, 2015). Samples were reconstituted in 200 μl of 80% (v/v) methanol and filtrated (PTFE, 0.22 μm; Anpel), then analyzed using an LC-ESI-MS/MS system (HPLC, Shim-pack UFLC SHIMADZU CBM30A system, http://www.shimadzu.com.cn/; MS, Applied Biosystems 6500 Triple Quadrupole, http://www.appliedbiosystems.com.cn/). A Waters Acquity UPLC BEH C_18_ column (2.1 mm×100 mm×1.8 µm) was used. The instrument was operated, and multiple reaction monitoring (MRM) transitions were assessed (Li *et al.*, 2017). For all samples, peak areas for endogenous and labeled GAs were compared and combined with 200 mg of powdered sample to calculate ng g^–1^.

### GA treatment

For application of GA to young plants growing on soil, gibberellic acid (GA_4_, Sangon Biotech) were first dissolved in absolute ethanol and then diluted with the nutrient solution (Miracle-Gro^®^ Pour & Feed Plant Food, Item #1006002). A solution of 10^–5^ M GA_4_ was sprayed on the plants twice a week starting 14 d post-germination. After 2 weeks of treatment, the treated plants were compared with the control plants (treated with the same solution without GA_4_).

### Dual-luciferase transient expression assay

The *SlKS5* and *SlGA20ox2* promoters were cloned into the pGreenII 0800-LUC double-reporter vector, while *SlbHLH95* was cloned into the pGreenII 62-SK vector effector, as described by Hellens *et al.* (2005). The constructed reporter and effector plasmids were transiently expressed in tobacco (*N. benthamiana*) leaves as described by Hellens *et al.* (2005). A dual-luciferase assay kit (Promega) was used to analyze the transient expression in tobacco leaves on the third day after infiltration. Absolute firefly luciferase (LUC) reporter and renilla luciferase (REN) values were measured in a Luminoskan Ascent Microplate Luminometer (Thermo Scientific) according to the manufacturer’s instructions, with a 5 s delay and 15 s integrated measurements. The transcriptional activation activity of SlbHLH95 on the promoters of GA biosynthesis-related genes was indicated by the ratio of LUC to REN. At least six biological repeats were conducted.

### Yeast one-hybrid assays

The full-length cDNA sequence of *SlbHLH95* was amplified and inserted into the pDEST22 vector containing the GAL4 activation domain (AD) to form the construct pDEST22-SlbHLH95. Three tandem copies of the E-box from *SlKS5* and *SlGA20ox2* promoters and the promoter fragment were each cloned into the pHis-Leu2-GW vector. Various combinations of pDEST22-SlbHLH95 and different promoters were co-transformed into yeast strain Y187. Empty pDEST22 with the two promoters was used as a negative control. The transformants were cultivated on SD/-Trp/-Leu medium for 3 d. DNA–protein interactions were determined by the growth of clones on SD/-Trp/-Leu/-His with 10 mM 3-AT (3-amino-1,2,4-triazole).

## Results

### Identification of SlbHLH95 as a putative regulator of tomato trichome formation

**Fig. 1.**
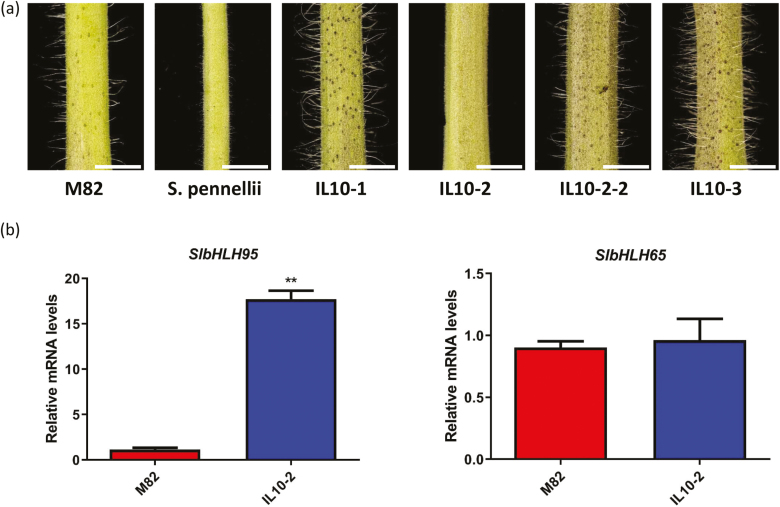
Different trichome phenotypes displayed by domesticated tomato (*S. lycopersicum*), a wild relative (*S. pennellii*), and introgression lines. (a) Introgression line IL10-2 showed altered trichome density in stem compared with the parental line M82. The white bars represent 1 cm. IL10-1, IL10-2, IL10-2-2, and IL10-3 are different introgression lines. (b) RT–qPCR analysis of mRNA levels of *SlbHLH95* and *SlbHLH65* in M82 and the introgression line IL10-2. Values are means ±SD. Statistical significance was determined by Student’s *t*-test: **0.001<*P*<0.01.

Phenotyping of the *S. pennellii*×*S. lycopersicum* (cv. M82) introgression population identified one specific introgression line (IL) on chromosome 10 (IL10-2) showing altered trichome density in the stem compared with the M82 control parental line (Fig. 1a). This suggested the presence of a putative trichome regulatory gene(s) in the replacement region specific to the IL10-2 line. The search for putative genes present in the *S. pennellii* genome region introgressed in IL10-2 identified 56 potential gene units resident in this region, and among these two were predicted to putatively encode bHLH TFs (*Solyc10g079050*, *SlbHLH95* and *Solyc10g079070*, *SlbHLH65*; Supplementary Table S1). Considering that *bHLH* family genes have been shown to regulate trichome formation in Arabidopsis and given that *SlbHLH95* displayed 15 times higher expression at the transcript level in stems of IL10-2 than in M82 (Fig. 1b), whereas *SlbHLH65* exhibited similar expression in the two tomato accessions (Fig. 1b), we postulated that *SlbHLH95* represents a realistic candidate gene underpinning the observed trichome phenotype. We therefore sought to address the functional significance of *SlbHLH95* in the regulation of trichome formation in tomato.

**Fig. 2.**
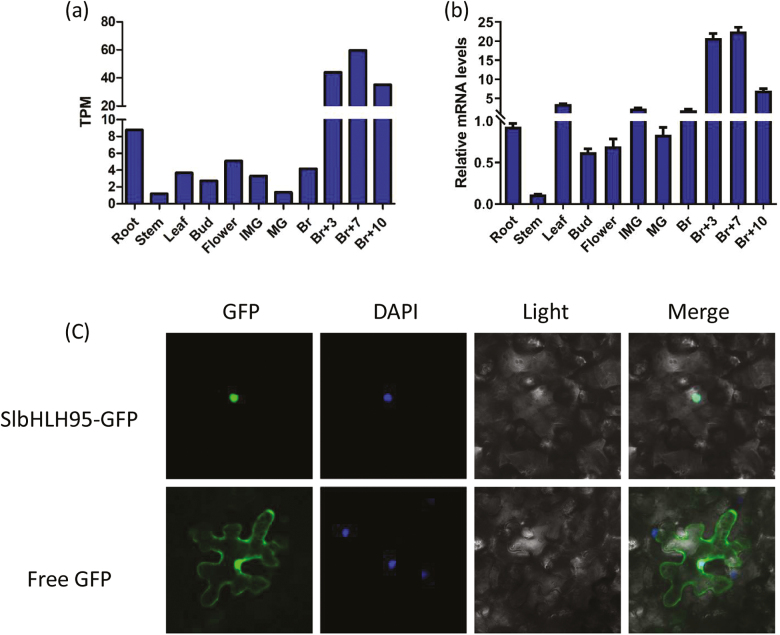
Expression pattern of *SlbHLH95* and subcellular localization of SlbHLH95 protein. (a) Expression levels of the *SlbHLH95* gene in different tissues. TPM, transcripts per kilobase of exon model per million mapped reads. (b) Relative mRNA levels of *SlbHLH95* in different tissues. Accumulation of *SlbHLH95* transcripts was assessed by RT–qPCR in root, stem, leaf, flower, mature green fruit (MG), breaker fruit (Br), 3 d post-breaker fruit (Br+3), and 7 d post-breaker fruit (Br+7). The relative mRNA levels of *SlbHLH95* in the root were standardized to 1.0, referring to the *SlActin* gene as the internal control. Values are means ±SD of three biological replicates. (c) GFP-tagged fusion proteins were transiently expressed under the control of the 35S promoter in tobacco cells after transfection.

RNA-Seq and RT–qPCR studies revealed that *SlbHLH95* was constitutively expressed in all tissues tested, with the highest transcript levels observed in ripe fruit and the lowest in stems where trichomes are present at high density (Fig. 2a, b), indicating that SlbHLH95 expression may negatively correlate with the presence of trichomes in the tissue. Confocal laser scanning microscopy analysis showed that SlbHLH95 strictly localized in the nucleus (Fig. 2c), consistent with its putative TF activity.

### Overexpression of *SlbHLH95* results in reduced trichome formation

**Fig. 3.**
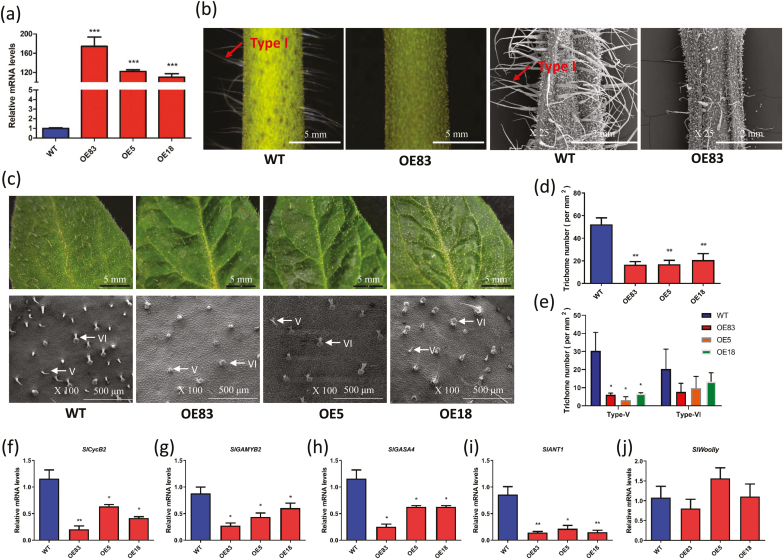
*SlbHLH95* overexpression lines show reduced trichome number on stems and leaves. (a) RT–qPCR analysis of *SlbHLH95* transcripts in *SlbHLH95-OE* lines. (b) Light microscopy and SEM observation of trichomes on stems. Arrows show type I trichomes. (c) Light microscopy and SEM observation of leaf trichomes. Observations were made on the adaxial surface of leaves at the same stage. Arrows show type V and type VI trichomes. (d) Density of trichomes on adaxial leaves from WT and *SlbHLH95-OE* plants. (e) The densities of the non-glandular type V trichome and the glandular type VI trichome are shown for the adaxial leaves. (f–j) RT–qPCR relative expression of trichome-associated genes in WT and *SlbHLH95-OE* lines in leaves at 4 weeks old. Values are means ±SD. Statistical significancewas determined by Student’s *t*-test: *0.01<*P*<0.05; **0.001<*P*<0.01; ***P<0.001. *OE83*, *OE5*, and *OE18* are three *SlbHLH95-OE* independent lines.

Since the expression level of *SlbHLH95* is very low in stems showing a high density of trichomes, we generated tomato overexpressing lines to uncover the role of this TF in trichome formation. Twelve *35S::SlbHLH95* independent homozygous lines were obtained that showed similar phenotypes. Three lines exhibiting 100- to 120-fold increases in the *SlbHLH95* transcript level were selected for further studies (Fig. 3a). *SlbHLH95-OE* plants exhibited a dramatic reduction in trichome number in stems compared with WT plants. Both light microscopy and SEM analyses showed that the decrease in trichome number mainly affected type I trichomes (Fig. 3b). Moreover, these analyses also revealed that the total trichome number was significantly decreased in leaves due to the decrease of type V trichomes (Fig. 3c–e) and in 7-day-old fruit of *SlbHLH95-OE* lines (Supplementary Fig. S1a, b). To gain insight into the molecular features underlying the reduced trichome phenotype, we assessed by RT–qPCR the transcript accumulation of genes known to be involved in trichome formation. *SlCycB2*, a B-type cyclin gene, shown to regulate tomato trichome formation, was significantly down-regulated in the *SlbHLH95-OE* lines (Fig. 3f). Other trichome-related genes, such as *SlGAMYB2*, *SlGASA4*, and *SlANT1*, also displayed a significant down-regulation in *SlbHLH95-OE* plants (Fig. 3g–i). In contrast, the transcript levels of *SlWoolly*, another gene reported to control trichome formation, showed no significant alteration (Fig. 3j).

### *SlbHLH95* overexpression lines display multiple vegetative and reproductive phenotypes

**Fig. 4.**
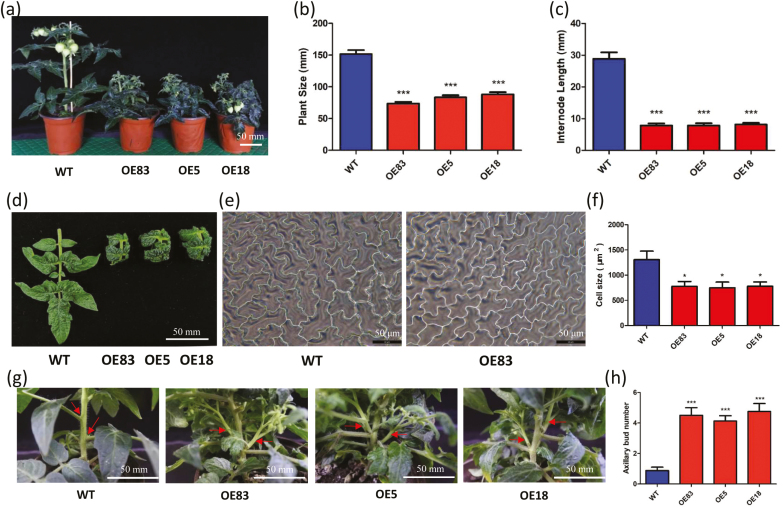
Vegetative phenotypes of *SlbHLH95* overexpression lines. (a) Dwarf phenotype of *SlbHLH95-OE* plants. Photographs were taken at 80 d after germination. (b) Reduced plant size of 80-day-old *SlbHLH95-OE* plants. Values are means ±SD (*n*≥15) of three replicates. (c) Shorter internodes of 80-day-old *SlbHLH95-OE* plants. Values are means ±SD (*n*≥15) of three replicates. (d) Leaf morphology of *SlbHLH95-OE* plants. (e) Light microscopy observation of a leaf epidermal cell in WT and *SlbHLH95-OE* plants. (f) Epidermal cell size in WT and *SlbHLH95-OE* leaves. *0.01<*P*<0.05 (Student’s *t*-test). *OE83*, *OE5*, and *OE18* are three independent *SlbHLH95-OE* lines. (g) Axillary bud phenotype of *SlbHLH95-OE* plants. Photographs were taken at the 8-week-old stage. (h) *SlbHLH95-OE* plants have more axillary buds than the WT. Values are the mean ±SD (*n*≥15) of three replicates. ***P < 0.001, (Student’s *t*-test).

In addition to the reduced trichome formation, *SlbHLH95-OE* lines exhibited a stunted phenotype from early developmental stages with a severe reduction in plant size (Fig. 4a). The average height of *SlbHLH95-OE* adult plants was less than half of that of WT plants due to shorter internodes (Fig. 4b, c). Leaf morphology was dramatically altered in the *SlbHLH95-OE* lines, with twisted leaf margins and wrinkled lamina, and leaf size was significantly reduced due to smaller epidermal cells (Fig. 4d–f). In addition, *SlbHLH95-OE* transgenic plants exhibited more axillary buds than WT plants (Fig. 4g). Unlike the WT that produced 1–2 axillary buds, *SlbHLH95-OE* lines had 3–5 axillary buds per plant at the 8-week-old stage (Fig. 4h).

**Fig. 5.**
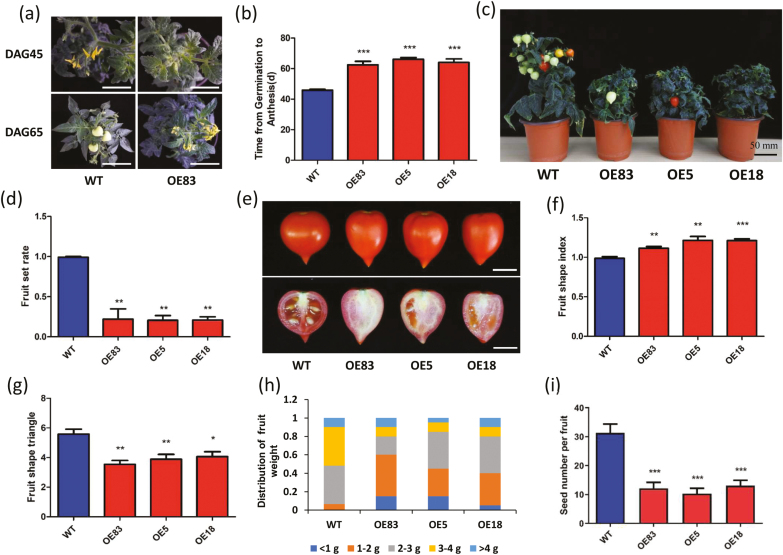
Reproductive development was dramatically altered in *SlbHLH95-OE* lines. (a, b) Delayed flowering of *SlbHLH95-OE* plants compared with the WT. DAG, days after germination. The white bars represent 5 cm. (c, d) Reduced fruit set in *SlbHLH95-OE* plants. (e) Altered fruit shape in *SlbHLH95-OE* plants. The white bars represent 1 cm. (f) Fruit shape index as the ratio of length to width in three representative independent lines. (g) Fruit shape triangle as the ratio of proximal end width to distal end width in three representative independent lines. (h) Distribution of fruit weight in WT and *SlbHLH95-OE* lines. *n*=60 fruits in WT and transgenic lines. (i) Seed number per fruit in WT and *SlbHLH95-OE* lines. *n*=60 fruits in WT and transgenic lines. Values are means ±SD. Statistical significance determined by Student’s *t*-test: *0.01<*P*<0.05; **0.001<*P*<0.01; ***P<0.001.

*SlbHLH95-OE* plants also showed large alterations in reproductive development. The time from germination to anthesis was delayed by ~20 d compared with the reference WT plants (Fig. 5a, b). Remarkably, although *SlbHLH95-OE* lines produced normal flower buds, the majority of these never reach the anthesis stage (Supplementary Fig. S2a), which is reminiscent of phenotypes due to a defect in GA (Nester and Zeevaart, 1988). Furthermore, the *SlbHLH95-OE* lines exhibited a dramatic reduction in fruit set, leading to markedly lower fruit number per plant (Fig. 5c). The fruit set rate was 15–20% in the *SlbHLH95-OE* lines, while 95% of the flowers successfully set fruit in WT plants grown under the same cultivation conditions (Fig. 5d). Cross-fertilization assays showed that pollen viability and female organ fertility were not affected in overexpressing lines (Supplementary Fig. S2b).

Although fruits of *SlbHLH95-OE* lines showed no significant difference compared with WT lines with respect to fruit development and ripening (Supplementary Fig. S2c, d), the fruit were more elongated with increased fruit shape index and decreased fruit shape triangle compared with the WT (Fig. 5e–g). The average fruit weight was also reduced, with a higher proportion of small fruits in *SlbHLH95-OE* lines (Fig. 5h). Moreover, the number of seeds per fruit was significantly reduced in *SlbHLH95-OE* lines (Fig. 5i).

### Genome-wide transcriptomic profiling of *SlbHLH95-OE* lines

To further unveil the molecular basis of the phenotypes observed in *SlbHLH95-OE* lines, we performed a global gene expression profiling of 4-week-old leaves with three biological replicates. The number of raw reads ranged from 43 to 55 million with an error rate of ~0.02%, producing 6.11–7.84 G clean bases (Supplementary Data S1). On average, 96% of these reads were mapped to the ITAG-2.4 tomato reference genome, yielding 38–49 million unique mapping reads based on the sample examined (Supplementary Data S2). The expression values, indicated by fragments per kilobase of transcript per million mapped fragments (FPKM) (Supplementary Data S3), showed high correlations (Pearson correlation coefficient >0.97) among biological replicates (Supplementary Data S4) (Trapnell *et al.*, 2010). Distribution plots showed that the distribution of the normalized expression levels was comparable between WT and OE lines (Supplementary Fig. S3).

**Fig. 6.**
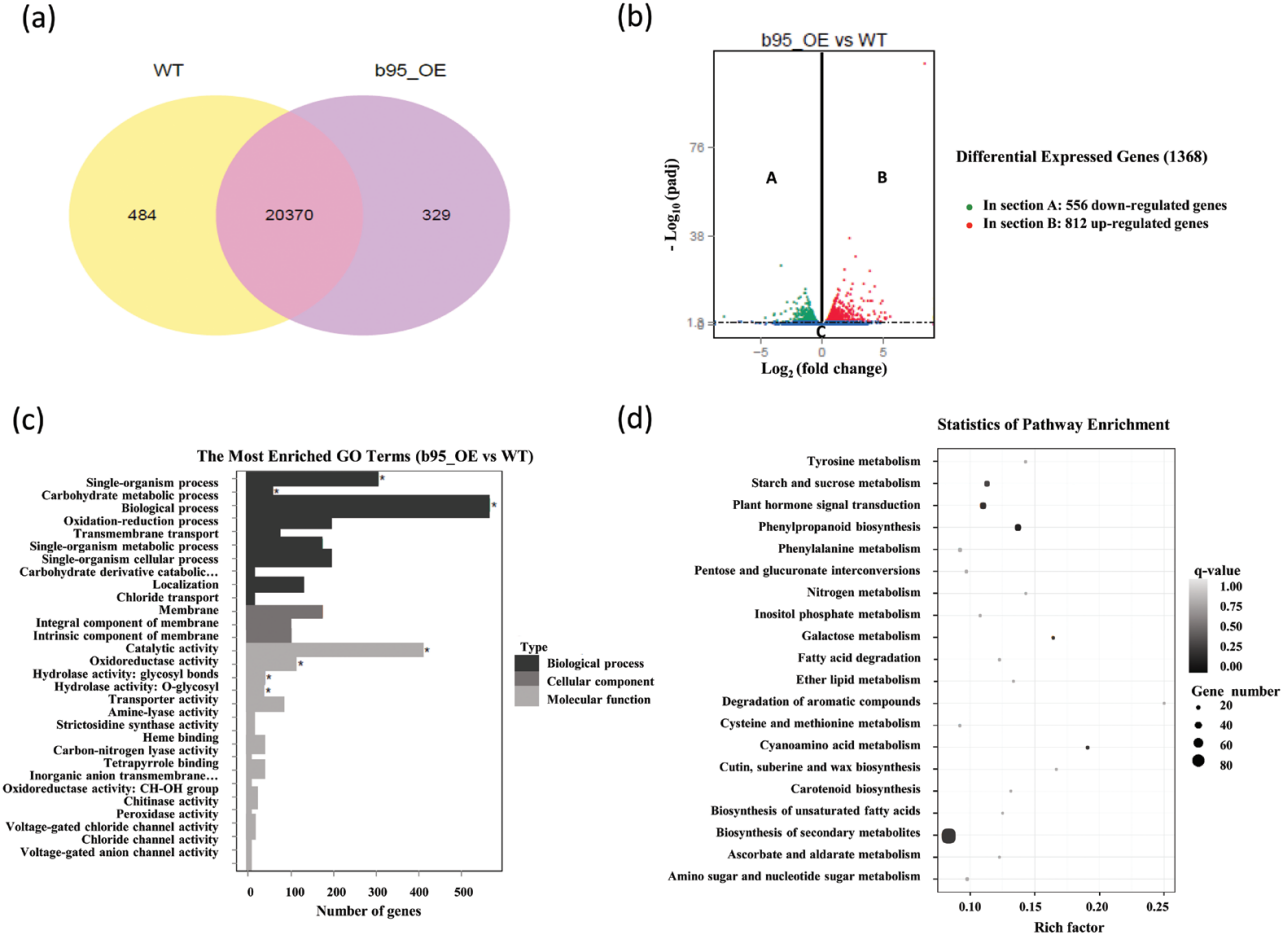
RNA-Seq profiling of *SlbHLH95-OE* lines. (a) Venn diagram of the numbers of expressed genes in WT and *SlbHLH95-OE* lines. (b) Volcano plot showing the DEGs between two different libraries. *q*<0.05 was used as the threshold to determine the significance of DEGs. Dots in section A show down-regulated genes, dots in section B represent up-regulated genes, and dots below the dash-dotted line (section C) indicate transcripts that did not change significantly in the *SlbHLH95-OE* library compared with the WT. (c) GO enrichment analysis of DEGs between *SlbHLH95-OE* and the WT. The most enriched GO terms are shown. Asterisks indicate significantly enriched GO terms (*q*<0.05). (d) KEGG pathway enrichment analysis of DEGs between *SlbHLH95-OE* and the WT. The left *y*-axis shows the KEGG pathway. The *x*-axis shows the Rich factor. A high *q*-value is represented by light gray, and a low *q*-value is represented by black (*q*<0.05).

A total of 20 854 and 20 699 genes were expressed (averaged FPKM ≥1) in WT and OE lines, respectively. Among all these genes, 484 and 329 genes were uniquely expressed in WT and OE lines, respectively (Fig. 6a). A total of 1368 DEGs were identified between the WT and OE lines, of which 812 were up-regulated and 556 down-regulated in *SlbHLH95-OE* lines (Fig. 6b). The putative functions of the DEGs were predicted using GO analysis. The DEGs were categorized into three main categories: biological process; molecular function; and cellular component (Fig. 6c; Supplementary Data S5). Using the KEGG annotation pathway for the DEGs of *SlbHLH95-OE* versus the WT, those related to ‘Biosynthesis of secondary metabolites’, ‘Plant hormone signal transduction’, and ‘Phenylpropanoid biosynthesis’ were over-represented (Fig. 6d; Supplementary Data S6). Consistent with the reduced trichome number, most of the genes previously reported to regulate trichome formation in tomato were down-regulated in the *SlbHLH95-OE* lines (Supplementary Fig. S4).

Considering that the phytohormone GA was reported to regulate trichome formation in Arabidopsis and that the *SlbHLH95-OE* lines displayed GA-deficient phenotypes such as reduced plant size, reduced trichome number, increased axillary buds, delayed flowering time, and reduced fruit set, we analyzed the expression of GA-related genes. Interestingly, among the top 20 DEGs that show down-regulation, we found two GA biosynthesis genes. Indeed, (–)-ent-kaurene synthase (*SlKS5*, *Solyc03g006550*) and gibberellin 20-oxidase-2 (*SlGA20ox2*, *Solyc06g035530*) showed significantly lower expression at the transcript level in *SlbHLH95-OE* lines (Table 1), suggesting that GA content might be decreased in *SlbHLH95-OE* plants.

**Table 1.** Top 20 down-regulated genes in *SlbHLH95-OE* lines

Solyc number	Log_2_fold	Description
Solyc12g056690	–7.9933	Serine/threonine-protein phosphatase 7 long form homolog
**Solyc03g006550**	**–4.6366**	**(–)-ent-kaurene synthase (*SlKS5*)**
Solyc10g085240	–3.9082	UDP-glucosyltransferase
Solyc06g009240	–3.8717	Cation/H(+) antiporter 15
Solyc07g006560	–3.6676	Hypersensitive response assisting protein
Solyc08g080710	–3.3537	Carboxyl-terminal peptidase
Solyc06g083470	–3.0144	Tropinone reductase-like protein 16
Solyc01g094870	–2.9991	MRNA clone RAFL21-92-I07
Solyc04g005600	–2.8636	MYB transcription factor
Solyc11g021060	–2.7435	Proteinase inhibitor
Solyc03g121680	–2.5768	β-Fructofuranosidase
Solyc06g075460	–2.5287	TGF-β receptor type I/II extracellular region
Solyc02g077300	–2.2942	Peroxidase 73
Solyc01g108610	–2.2776	Cyclin-dependent kinase inhibitor 12
**Solyc06g035530**	**–2.1946**	**Gibberellin 20-oxidase-2 (*SlGA20ox2*)**
Solyc09g075790	–2.1653	Long-chain fatty acid CoA ligase
Solyc03g114830	–2.1535	MADS box transcription factor
Solyc09g072700	–2.1518	Peroxidase 57
Solyc04g014410	–2.1116	Serine/threonine protein kinase-like
Solyc04g056450	–2.0013	Cyclopropane-fatty-acyl-phospholipid synthase

All the genes were allocated a *q*-value <0.05.

### *SlbHLH95-OE* lines exhibit decreased gibberellin contents

**Fig. 7.**
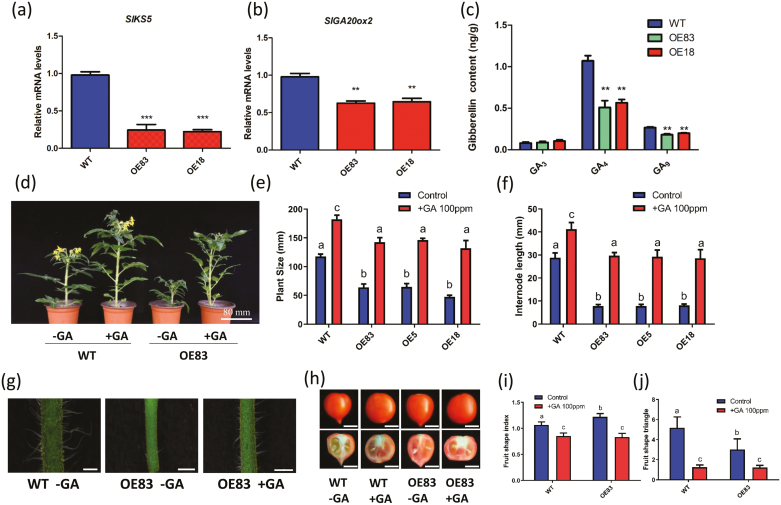
Application of exogenous GA rescued the dwarf phenotype in *SlbHLH95-OE* plants. (a, b) Relative expression of GA biosynthesis-related genes in the WT and *SlbHLH95-OE* lines in leaves at 4 weeks old. Statistical significance was determined by Student’s *t*-test: **0.001<*P*<0.01; ***P<0.001. (c) GA content was decreased in *SlbHLH95-OE* plants. **0.001< *P*<0.01 (Student’s *t*-test). (d) GA application rescued *SlbHLH95-OE* plant size. (e) Plant size of *SlbHLH95-OE* compared with WT plants after GA application. Values are means ±SD (*n*≥15) of three replicates. The error bars and letters in the graph indicate the SEs among samples and significant differences in plant size evaluated by Tukey’s test (α<0.05). (f) Internode length in *SlbHLH95-OE* plants treated with exogenous GA compared with that of WT plants. Values are the mean ±SD (*n*≥15) of three replicates. (g) Trichome density was partially rescued by exogenous GA application in *SlbHLH95-OE* lines. The white bars represent 2 mm. (h) Fruit shape rescued by exogenous GA application in *SlbHLH95-OE* lines. The white bars represent 1 cm. (i) The fruit shape index of GA_4_-treated and untreated plants. Values are means ±SD (*n*≥10) of three replicates. The error bars and letters in the graph indicate the SEs among samples and significant differences in plant size evaluated by Tukey’s test (α<0.05). (j) The fruit shape triangle of GA_4_-treated and untreated plants. Values are means ±SD (*n*≥10) of three replicates. The error bars and letters in the graph indicate the SEs among samples and significant differences in plant size evaluated by Dunnett’s T3 test (α<0.05).

Transcript accumulation corresponding to *SlKS5* and *SlGA20ox2* genes was further assessed by RT–qPCR, showing a significant decrease in *SlbHLH95-OE* lines compared with the WT (Fig. 7a, b). Assessing the GA content indicated that although GA_3_, the active GA form, displayed no change, GA_4_, the final product of the GA bioactive form, and its precursor GA_9_ were significantly decreased in *SlbHLH95-OE* plants (Fig. 7c). These results suggested that the decreased GA levels may account for the inhibition of trichome formation and for some of the vegetative and reproductive developmental alterations in *SlbHLH95-OE* lines.

To further investigate the contribution of the reduced GA levels to the phenotypes of low trichome density and altered vegetative and reproductive development in *SlbHLH95-OE* plants, we applied exogenous GA_4_ to 2-week-old plants since GA_4_ levels were found to be significantly decreased in the transgenic lines. The plant size of *SlbHLH95-OE* plants was fully rescued by GA_4_ treatment (Fig. 7d, e), with the internode length becoming comparable with those of untreated WT plants (Fig. 7f). Moreover, trichome density was enhanced in *SlbHLH95-OE* lines treated with exogenous GA_4_ (Fig. 7g). Interestingly, the fruit shape of GA-treated plants became more round compared with untreated *SlbHLH95-OE* plants (Fig. 7h) and, accordingly, both fruit shape index and fruit shape triangle were significantly decreased (Fig. 7i, j). These results indicated that the reduced trichome density, dwarf plant phenotype, and elongated fruit shape displayed by *SlbHLH95-OE* lines are likely to be due to the decreased GA levels.

### SlbHLH95 directly regulates *SlGA20ox2* and *SlKS5* via binding to their promoters

**Fig. 8.**
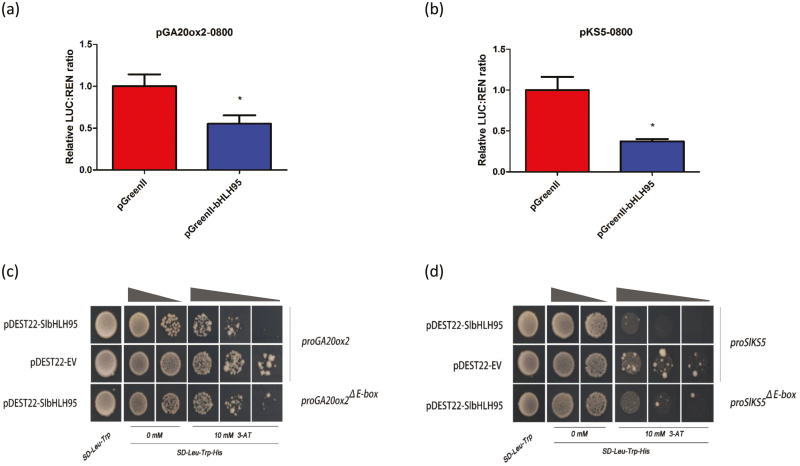
SlbHLH95 represses the expression of *SlGA20ox2* and *SlKS5* through direct binding to their *cis*-regulatory elements. (a, b) Dual-luciferase reporter assay. Interactions of SlbHLH95 with the promoters of *SlGA20ox2* and *SlKS5* were assessed *in vivo* by transient assays in tobacco leaves. The trans-repression ability of SlbHLH95 is indicated by the LUC:REN ratio. Each value represents the mean of six biological replicates, and vertical bars represent the SE. Significant difference (Student’s *t*-test): *0.01<*P*<0.05; LUC, firefly luciferase; REN, renilla luciferase. (c, d) SlbHLH95 represses the activity of the *SlGA20ox2* and *SlKS5* promoters as indicated by yeast one-hybrid assays. Yeast cells were co-transformed with a bait vector, containing a promoter fragment (*proSlGA20ox2* and mutated *proSlGA20ox2*; *proSlKS5* and mutated *proSlKS5*) fused to a *HIS2* reporter gene, and a prey vector, containing SlbHLH95 fused to the GAL4 activation domain. Cells were grown in liquid medium to OD_600_ of 0.1 (10^–1^) and diluted in a 10× dilution series (10^–2^ to 10^–3^). Of each dilution, 5 µl was spotted on media selecting for both plasmids (SD-Leu-Trp) and selecting for interaction (SD-Leu-Trp-His), supplemented with 10 mM 3-AT to suppress background growth and test the strength of the interaction.

The decreased GA content together with the down-regulated expression of *SlKS5* and *SlGA20ox2* in *SlbHLH95-OE* lines motivated the investigation of a possible regulation of *SlKS5* and *SlGA20ox2* transcription by SlbHLH95. An *in silico* search for typical regulatory motifs in the *SlKS5* and *SlGA20ox2* promoter sequences revealed the presence of conserved E-box (CANNTG) and E-box like *cis*-elements known as putative targets of bHLH type TFs (Supplementary Table S2). To examine whether SlbHLH95 could repress the transcription of a reporter gene driven by the *SlKS5* and *SlGA20ox2* promoters, transactivation assays using the dual-luciferase reporter system were performed. The dual luciferase reporter plasmids harboring the *SlKS5* and *SlGA20ox2* promoters that contain the E-box-like motif were fused to the LUC reporter, and the REN reporter driven by the 35S promoter was used as an internal control. The expression of SlbHLH95 resulted in reduced activity of both *SlKS5* and *SlGA20ox2* promoters, indicating that SlbHLH95 exerts a negative regulation on the transcription of the two GA biosynthesis genes (Fig. 8a, b).

To assess whether SlbHLH95 directly binds to the *SlKS5* and *SlGA20ox2* promoters, we then expressed SlbHLH95 fused to the GAL4 AD in a yeast one-hybrid system to test its ability to bind to the two promoters fused to the HIS2 reporter. The results showed that SlbHLH95 significantly reduced yeast growth compared with the control (Fig. 8c, d), whereas when challenged with mutated E-box sequences SlbHLH95 failed to significantly affect yeast growth (Fig. 8c, d), indicating that SlbHLH95 represses the activities of *SlKS5* and *SlGA20ox2* promoters via binding to the E-box *cis*-elements. Collectively, these data indicate that SlbHLH95 acts as a negative regulator of GA biosynthesis through direct binding to the E-box motif within the *SlKS5* and *SlGA20ox2* promoters. This is probably the causal factor leading to reduced trichome formation and other vegetative and reproductive developmental phenotypes.

## Discussion

Development of unicellular non-glandular trichomes is known to be regulated by a network of TFs in Arabidopsis (Schellmann and Hulskamp, 2005; Yang and Ye, 2013; Qi *et al.*, 2014), and phytohormones such as GA, JA, and CK were reported to play an essential role in this process acting in a TF complex-dependent manner (Pattanaik *et al.*, 2014). In contrast, less is known about regulatory networks that control the development of trichomes in crop species. Our present study shows that the TF SlbHLH95 is involved in the regulation of trichome development in tomato through the negative regulation of GA biosynthesis. Overexpression of *SlbHLH95* resulted in reduced trichome density, reduced plant size, increased axillary buds, delayed flowering time, poor fruit set, and elongated fruit shape that resemble GA-deficient phenotypes. Supporting this notion, GA content was significantly reduced in *SlbHLH95-OE* lines due to lower expression of two key GA biosynthesis genes, *SlGA20ox2* and *SlKS5*. The role of GA is further sustained by the rescue of the phenotypes by exogenous treatment of *SlbHLH95-OE* lines with the hormone. Yeast one-hybrid and transient expression assays showed that SlbHLH95 regulates the expression of *SlGA20ox2* and *KS5* via binding to their promoters. Altogether, these data validate the link between *SlbHLH95*, GA, and trichome formation in tomato.

It was reported that the bHLH TFs GL3 and EGL3 regulate trichome development in a partially redundant manner (Payne *et al.*, 2000; Zhang, 2003), and other members of the *bHLH* gene family, such as *AtMYC1* and *TRANSPARENT TESTA 8* (*TT8*), have also been shown to positively regulate trichome development in Arabidopsis (Maes *et al.*, 2008; Zhao *et al.*, 2012). The present study indicates that bHLH-type TFs are not only essential for trichome formation in Arabidopsis, but they are also involved in this process in tomato. This finding is consistent with recent reports on the role of *SlMYC1*, another *bHLH* family gene, that positively regulates type VI glandular trichome formation in tomato (Xu *et al.*, 2018). Interestingly, unlike other reported bHLH TFs, such as SlMYC1 which play a positive role in type VI trichome development (Payne *et al.*, 2000; Zhang, 2003; Maes *et al.*, 2008; Zhao *et al.*, 2012; Shangguan *et al.*, 2016; Xu *et al.*, 2018), overexpression of *SlbHLH95* in tomato resulted in dramatically reduced type I trichomes on stems and significantly fewer type V trichomes on leaves.

Mutation in the *C2H2 ZFP* gene (*Solyc10g078970*) has been suggested to account for the glabrous phenotype of IL10-2 and the *hair absent* (*h*) mutant LA3172 (Chang *et al.*, 2018). Our results demonstrate that the TF gene *SlbHLH95* (*Solyc10g079050*), mapping to the same region on chromosome 10 as *C2H2 ZFP*, also plays an active role in tomato trichome formation. Interestingly, in addition to the glabrous phenotype, overexpression of *SlbHLH95* in Micro-Tom induced pleiotropic alterations associated with GA deficiency, including reduced plant size, more axillary buds, lower fruit set, and elongated fruit shape, which are absent in *S. pennellii* and IL10-2, probably due to the different expression levels of *SlbHLH95* in these lines. It is also worth pointing out that the cultivar used in this study is Micro-Tom. Although the Micro-Tom cultivar is a brassinosteroid mutant and its trichome formation might be different from that other tomato cultivars, it has been shown to be a good model to study the regulation of trichome formation (Campos *et al.*, 2009; Zhang *et al.*, 2015).

In Arabidopsis, GAs play an essential role in unicellular trichome development as shown by the GA-deficient mutant *ga1-3* that displays glabrous leaves, and treatment with exogenous GAs restores trichome formation (Perazza *et al.*, 1998). Likewise, mutations of GA signaling-related genes can also affect trichome development in Arabidopsis (Chien and Sussex, 1996; Dill and Sun, 2001; Gan *et al.*, 2007). GA transport and distribution were recently reported to be important in trichome formation (Matías-Hernández *et al.*, 2016). While it is now established that GAs promote unicellular trichome initiation and development in Arabidopsis, the putative role of GAs in trichome formation in a crop species such as tomato remains largely unknown. Our data show that GA content was significantly decreased in *SlbHLH95* overexpression plants along with the down-regulation of two key GA biosynthesis genes, consistent with the displayed GA-deficient phenotypes. Moreover, application of exogenous GA to these plants restored several WT phenotypes including trichome formation, plant size, and axillary bud number, thus suggesting that similar to the situation for unicellular trichomes in Arabidopsis, GA may also play an important role in the formation of different types of trichomes in tomato. Interestingly, several TF genes including *SlIAA15*, *SlARF3*, and *SlMYC1* reported to positively regulate trichome formation in tomato (Deng *et al.*, 2012; Zhang *et al.*, 2015; Xu *et al.*, 2018) showed a down-regulation tendency in *SlbHLH95-OE* lines (Supplementary Fig. S4). In addition, a number of *MYB*, *bHLH*, and *WDR* gene family members also showed altered transcript levels in *SlbHLH95-OE* plants (Supplementary Data S7). Taken together, these results suggest that the way in which GAs regulate trichome formation in tomato might be through modulating the expression of key trichome regulators as in Arabidopsis.

In Arabidopsis, several TFs have been shown to affect GA content via the regulation of GA biosynthesis genes, such as the MADS domain protein AGAMOUS that promotes the expression of *AtGA3ox1* in developing ﬂowers (Gomez-Mena, 2005), the bHLH TF INDETERMINATE, reported to directly regulate *AtGA3ox1* in the valve margins and septum of the Arabidopsis silique (Arnaud *et al.*, 2010), and the MADS protein SVP which delays flowering by repressing GA biosynthesis via reducing expression of *AtGA20ox2* (Andrés *et al.*, 2014). In contrast, very little is known about the direct regulation of GA biosynthesis genes in tomato. Our data reveal that SlbHLH95 regulates the expression of two key GA biosynthesis genes, *SlGA20ox2* and *SlKS5*, via direct binding to their promoters, thus extending our understanding of the regulation of GA biosynthesis in tomato. While the present data reveal that GA is involved in trichome development, additional studies are needed to uncover the molecular mechanisms underlying GA regulation of trichome formation in tomato. In particular, it remains to be investigated whether trichome formation involving SlbHLH95 is also mediated by a complex similar to the MYB–bHLH–WDR complex described in Arabidopsis.

## Supplementary data

Supplementary data are available at *JXB* online.

Fig. S1. The glabrous phenotype exhibited by *SlbHLH95-OE* young fruit.

Fig. S2. Reproductive development of *SlbHLH95-* overexpressing lines.

Fig. S3. The gene expression distribution (FPKM) in WT and *SlbHLH95-OE* lines.

Fig. S4. Expression levels of trichome-related genes in WT and *SlbHLH95-OE* lines.

Table S1. Genes contained in the *S. pennellii* genome fragment introgressed in M82 to give rise to the IL10-2 line.

Table S2. Putative bHLH-binding *cis*-elements present in the promoter regions of *SlGA20ox2*, *SlKS5.*

Table S3. List of the primers used in the study.

Dataset S1. Quality assessment of sequencing data.

Dataset S2. Reads mapping to the reference genome.

Dataset S3. The expression levels of genes in SlbHLH95-OE and wild-type (WT) lines.

Dataset S4. Pearson correlation coefficient analysis.

Dataset S5. GO enrichment analysis of DEGs between SlbHLH95-OE (b95_OE) and WT lines.

Dataset S6. KEGG enrichment analysis of DEGs between SlbHLH95-OE (b95_OE) and WT lines.

Dataset S7. The expression levels of MYB, bHLH and WD40 family genes in SlbHLH95-OE and WT lines.

eraa114_suppl_Supplementary_Figure_S1_S4

eraa114_suppl_Supplementary_Table_S1_S3

eraa114_suppl_Supplementary_Dataset_S1_S7

## Data Availability

All relevant data are within the paper and its supplementary files.
