# Supplementary material for: Overexpression of bHLH95, a basic helix–loop–helix transcription factor family member, impacts trichome formation via regulating gibberellin biosynthesis in tomato
Source: J Exp Bot. 2020 Mar 5;71(12):3450–62. doi: 10.1093/jxb/eraa114 (PMC7475245; doi:10.1093/jxb/eraa114)
Supplement: eraa114_suppl_Supplementary_Figure_S1_S4 [file eraa114_suppl_supplementary_figure_s1_s4.pdf]

WT

OE83

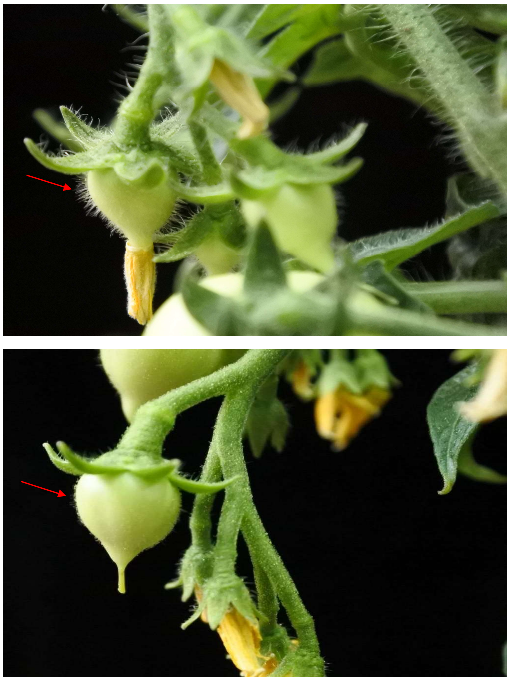

Figure S1. The glabrous phenotype exhibited by *SlbHLH95-OE* young fruit.

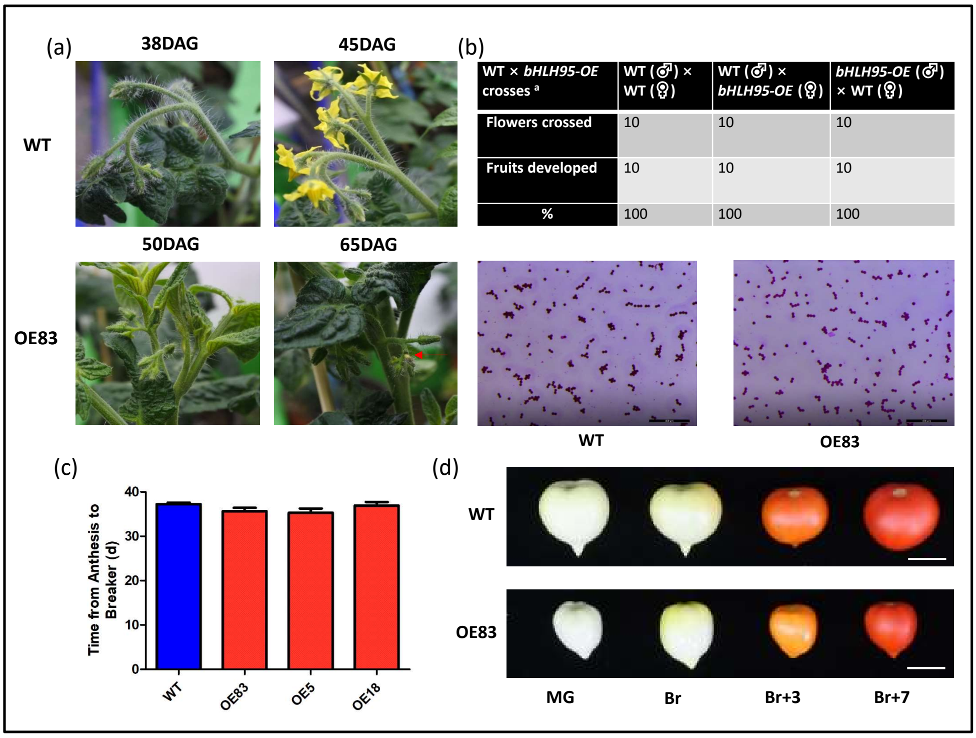

WT × *bHLH95-OE* crosses [a]	WT (♂) × WT (♀)	WT (♂) × *bHLH95-OE* (♀)	*bHLH95-OE* (♂) × WT (♀)
Flowers crossed	10	10	10
Fruits developed	10	10	10
%	100	100	100

Figure S2. Reproductive development phenotype observation.

(a) Abnormal development of flower bud in *SlbHLH95-OE* lines.

(b) Similar fertility between wild-type (WT) and *SlbHLH95-OE* lines. Cross-fertilization assay and Alexander's staining test. a: All flowers were emasculated and then crossed with the respective pollen.

(c) Time period from anthesis to the breaker stage (Br) in WT and *SlbHLH95-OE* lines.

(d) Different ripening stages in WT and *SlbHLH95-OE* lines. MG, mature green; Br, breaker stage; Br+3, 3 days post-Br stage; Br + 7, 7 days post-Br stage. The white bars represent 1 cm.

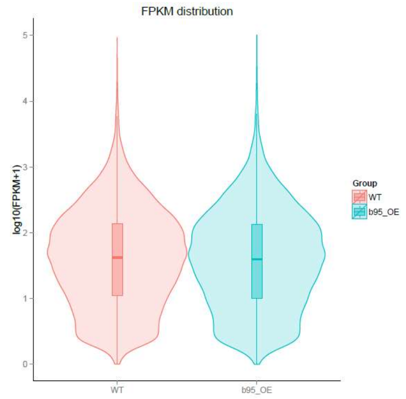

Figure S3. The gene expression distribution (FPKM) in wild-type (WT) and *SlbHLH95-OE* lines.

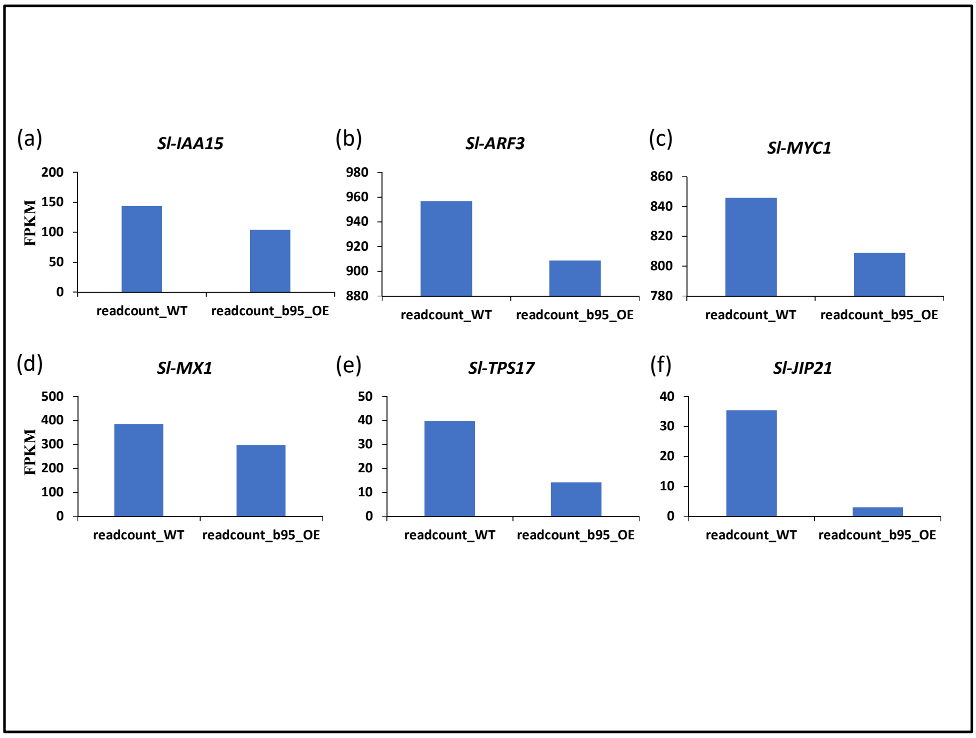

Figure S4. Expression levels of trichome related genes in WT and OE lines.
(a-e) Expression levels of trichome related genes in WT and OE lines. FPKM, Fragments Per Kilobase Million.
