# Supplementary material for: Overexpression of bHLH95, a basic helix–loop–helix transcription factor family member, impacts trichome formation via regulating gibberellin biosynthesis in tomato
Source: J Exp Bot. 2020 Mar 5;71(12):3450–62. doi: 10.1093/jxb/eraa114 (PMC7475245; doi:10.1093/jxb/eraa114)
Supplement: eraa114_suppl_Supplementary_Table_S1_S3 [file eraa114_suppl_supplementary_table_s1_s3.pdf]

Table S1. Genes specifically replaced by *S.pennellii* genome located in IL10-2

Gene	Gene description
Solyc10g078940	Receptor-like kinase (AHRD V1 ***- A7VM24_MARPO); contains Interpro domain(s) IPR002290 Serine/threonine protein kinase
Solyc10g078950	Glucose-repressible alcohol dehydrogenase transcriptional effector CCR4 (AHRD V1 **-- D3TNH5_GLOMM); contains Interpro domain(s) IPR005135 Endonuclease/exonuclease/phosphatase
Solyc10g078960	60S ribosomal protein L21-like protein (AHRD V1 ***- Q3HVK5_SOLTU); contains Interpro domain(s) IPR001147 Ribosomal protein L21e
Solyc10g078970	Zinc finger protein (AHRD V1 *-*- B6SZ06_MAIZE); contains Interpro domain(s) IPR007087 Zinc finger, C2H2-type
Solyc10g078980	Unknown Protein (AHRD V1)
Solyc10g078990	Zinc finger-like protein (AHRD V1 ***- Q9M2P1_ARATH); contains Interpro domain(s) IPR007087 Zinc finger, C2H2-type
Solyc10g079000	Transmembrane and coiled-coil domain-containing protein 1 (AHRD V1 ***- B0WCQ4_CULQU); contains Interpro domain(s) IPR008559 Protein of unknown function DUF841, eukaryotic
Solyc10g079010	Unknown Protein (AHRD V1)
Solyc10g079020	Cc-nbs-lrr resistance protein (AHRD V1 ***- B9NCL4_POPTR)
Solyc10g079030	Ras-related protein Rab-25 (AHRD V1 ***- RAB25_RABIT); contains Interpro domain(s) IPR015595 Rab11-related
Solyc10g079040	Squalene synthase (AHRD V1 ***- O22107_SOYBN); contains Interpro domain(s) IPR006449 Farnesyl-diphosphate farnesyltransferase
**Solyc10g079050**	**BHLH transcription factor (AHRD V1 *-*- B6TXR4_MAIZE); contains Interpro domain(s) IPR011598 Helix-loop-helix DNA-binding**
Solyc10g079060	Squalene synthase (AHRD V1 ***- Q9XF02_CAPAN); contains Interpro domain(s) IPR006449 Farnesyl-diphosphate farnesyltransferase
**Solyc10g079070**	**Transcription factor (AHRD V1 *-*- D6MKM4_9ASPA); contains Interpro domain(s) IPR011598 Helix-loop-helix DNA-binding**
Solyc10g079080	Zinc finger A20 and AN1 domain-containing stress-associated protein 6 (AHRD V1 *-*- C1BNQ4_9MAXI); contains Interpro domain(s) IPR002653 Zinc finger, A20-type IPR000058 Zinc finger, AN1-type
Solyc10g079090	Chaperone protein dnaJ 6 (AHRD V1 ***- B6TT74_MAIZE); contains Interpro domain(s) IPR003095 Heat shock protein DnaJ
Solyc10g079100	Unknown Protein (AHRD V1)
Solyc10g079110	1-acyl-sn-glycerol-3-phosphate acyltransferase (AHRD V1 ***- Q2Z1Y8_PRUMU); contains Interpro domain(s) IPR002123 Phospholipid/glycerol acyltransferase
Solyc10g079120	Zinc finger CCCH domain-containing protein 30 (AHRD V1 **-- C3H30_ARATH); contains Interpro domain(s) IPR002110 Ankyrin
Solyc10g079130	Calcium-dependent protein kinase 4 (AHRD V1 ***- Q5EDD1_CAPAN); contains Interpro domain(s) IPR002290 Serine/threonine protein kinase
Solyc10g079140	Guanylate kinase (AHRD V1 ***- Q9FVC6_TOBAC); contains Interpro domain(s) IPR017665 Guanylate kinase, sub-group
Solyc10g079150	Nuclear transcription factor Y subunit A-1 (AHRD V1 *-*- B6TAR1_MAIZE); contains Interpro domain(s) IPR001289 CCAAT-binding transcription factor, subunit B
Solyc10g079160	Os01g0617600 protein (Fragment) (AHRD V1 ***- Q0JL81_ORYSJ); contains Interpro domain(s) IPR001578 Peptidase C12, ubiquitin carboxyl-terminal hydrolase 1
Solyc10g079170	Receptor like kinase, RLK
Solyc10g079180	Unknown Protein (AHRD V1); contains Interpro domain(s) IPR001932 Protein phosphatase 2C-related
Solyc10g079190	Pre-mRNA splicing factor (AHRD V1 *-*- C4QX20_PICPG); contains Interpro domain(s) IPR000504 RNA recognition motif, RNP-1

Solyc10g079200	Mitochondrial carrier protein (AHRD V1 ***- B1N662_SOLLC); contains Interpro domain(s) IPR018108 Mitochondrial substrate/solute carrier IPR001993 Mitochondrial substrate carrier
Solyc10g079210	Abscisic acid insensitive 8 homologue (AHRD V1 **-- A5A6Q2_WHEAT)
Solyc10g079220	Unknown Protein (AHRD V1); contains Interpro domain(s) IPR003441 No apical meristem (NAM) protein
Solyc10g079230	CM0545.450.nc protein (AHRD V1 ***- B0BLB7_LOTJA); contains Interpro domain(s) IPR004158 Protein of unknown function DUF247, plant
Solyc10g079240	Calmodulin binding protein (AHRD V1 *--- B6T589_MAIZE); contains Interpro domain(s) IPR000048 IQ calmodulin-binding region
Solyc10g079250	Protein recA (AHRD V1 ***- Q0F3T6_9PROT); contains Interpro domain(s) IPR013765 RecA IPR003593 ATPase, AAA+ type, core
Solyc10g079260	Os06g0301100 protein (Fragment) (AHRD V1 *-*- Q0DCQ4_ORYSJ); contains Interpro domain(s) IPR005349 Uncharacterised protein family UPF0136, Transmembrane
Solyc10g079270	Carbonic anhydrase family protein (AHRD V1 ***- D7MFG3_ARALY); contains Interpro domain(s) IPR018340 Carbonic anhydrase, CAH1-like
Solyc10g079280	Organic anion transporter (AHRD V1 **-* D7L9W2_ARALY); contains Interpro domain(s) IPR004853 Protein of unknown function DUF250
Solyc10g079290	Outer envelope membrane protein (AHRD V1 **-- Q31PR1_SYNE7); contains Interpro domain(s) IPR000184 Bacterial surface antigen (D15)
Solyc10g079300	Calcium-transporting ATPase 1 (AHRD V1 ***- Q7XBH9_CERRI); contains Interpro domain(s) IPR006408 ATPase, P-type, calcium-transporting, PMCA-type
Solyc10g079310	tRNA-splicing endonuclease subunit sen54 (AHRD V1 *-*- B6JWH8_SCHJY)
Solyc10g079320	Glucosyltransferase-5 (AHRD V1 *-** Q8S9A4_PHAAN); contains Interpro domain(s) IPR002213 UDP-glucuronosyl/UDP-glucosyltransferase
Solyc10g079330	UDP-glucosyltransferase HvUGT5876 (AHRD V1 *-*- D3WYW1_HORVD); contains Interpro domain(s) IPR002213 UDP-glucuronosyl/UDP-glucosyltransferase
Solyc10g079340	UDP-glucosyltransferase (AHRD V1 *-** B8QI32_9MAGN); contains Interpro domain(s) IPR002213 UDP-glucuronosyl/UDP-glucosyltransferase
Solyc10g079350	UDP-glucosyltransferase HvUGT5876 (AHRD V1 ***- D3WYW1_HORVD); contains Interpro domain(s) IPR002213 UDP-glucuronosyl/UDP-glucosyltransferase
Solyc10g079360	Transcription initiation factor IIB-2 (AHRD V1 ***- D7L8X6_ARALY); contains Interpro domain(s) IPR000812 Transcription factor TFIIB related
Solyc10g079370	Transcription initiation factor IIB-2 (AHRD V1 ***- D7L8X6_ARALY); contains Interpro domain(s) IPR000812 Transcription factor TFIIB related
Solyc10g079380	Heat stress transcription factor (AHRD V1 ***- D4QAU8_CARPA); contains Interpro domain(s) IPR000232 Heat shock factor (HSF)-type, DNA-binding
Solyc10g079390	Unknown Protein (AHRD V1)
Solyc10g079400	Gelsolin (Fragment) (AHRD V1 *--- C0PU67_SALSA); contains Interpro domain(s) IPR007122 Gelsolin
Solyc10g079410	Patatin-like phospholipase domain-containing protein c (AHRD V1 *--- B6K1H8_SCHJY); contains Interpro domain(s) IPR006689 ARF/SAR superfamily
Solyc10g079420	Calmodulin (AHRD V1 ***- B6TKX0_MAIZE); contains Interpro domain(s) IPR011992 EF-Hand type
Solyc10g079430	U3 small nucleolar RNA-associated protein 11 (AHRD V1 ***- B6TNQ8_MAIZE); contains Interpro domain(s) IPR007144 Small-subunit processome, Utp11
Solyc10g079440	FAD-dependent oxidoreductase family protein (AHRD V1 ***- D7LVK2_ARALY); contains Interpro domain(s) IPR006076 FAD dependent oxidoreductase
Solyc10g081860	Unknown Protein (AHRD V1)
Solyc10g081870	Digalactosyldiacylglycerol synthase (Fragment) (AHRD V1 ***- Q6DQ98_TOBAC)
Solyc10g081880	Pentatricopeptide repeat-containing protein (AHRD V1 ***- D7MTZ6_ARALY); contains Interpro domain(s) IPR002885 Pentatricopeptide repeat
Solyc10g081890	Aluminum-activated malate transporter (Fragment) (AHRD V1 *--- Q07DP9_AEGSP); contains Interpro domain(s) IPR006214 Uncharacterised protein family UPF0005

Solyc10g081900	Digalactosyldiacylglycerol synthase 1, chloroplastic (AHRD V1 ***- DGDG1_LOTJA); contains Interpro domain(s) IPR001296 Glycosyl transferase, group 1

Table S2. Putative bHLH binding *cis*-elements present in the promoter regions of *SlGA20ox2*, *SlKS5*. The genomic sequence corresponding to 3.0 kb upstream of the predicted translation start codon (ATG) was analyzed for the presence of known *cis*-acting elements.

**Gene**	**Motif**	**Position**	**Sequence**
**GA biosynthesis genes**			
SlGA20ox2	E-box	-2976	CAATTG
	E-box	-2842	CAAGTG
	E-box-like	-2823	CAATAG
	E-box	-2796	CAATTG
	E-box-like	-2771	CTCTTG
	E-box-like	-2755	CAAACG
	E-box-like	-2689	CTATTG
	E-box-like	-2658	CTATTG
	E-box-like	-2641	CCATTG
	E-box-like	-2627	CTTTTG
	E-box-like	-2436	CAAAAG
	E-box	-2418	CAATTG
	E-box-like	-2111	CTATTG
	E-box-like	-1796	CAAAGG
	E-box-like	-1783	CAAGAG
	E-box-like	-1766	CAATAG
	E-box-like	-1735	CAATAG
	E-box-like	-1704	CAATAG
	E-box-like	-1457	CACTCG
	E-box-like	-1392	CGTTTG
	E-box-like	-1222	CATCCG
	E-box-like	-1017	CTGGTG
	E-box-like	-970	CAAGAG
	E-box-like	-935	CAGTAG
	E-box-like	-686	CGTATG
	E-box-like	-554	CGAATG
	E-box-like	-179	CTCGTG
	E-box-like	-156	CATTGT
	E-box-like	-112	CTTATG
	E-box-like	-74	CCCTTG
	E-box-like	-46	CTCCTG
SlKS5	E-box	-2789	CATGTG
	E-box-like	-2777	CGTATG
	E-box-like	-2671	CAAGAG
	E-box-like	-2641	CTTATG
	E-box-like	-2619	CAATCG
	E-box	-2604	CACATG
	E-box-like	-2536	CATTCG
	E-box-like	-2525	CATACG
	E-box-like	-2502	CTTTTG
	E-box-like	-2369	CATTAG

E-box-like	-2190	CATAAG
E-box-like	-2143	CAAAAG
E-box-like	-2090	CGCTTG
E-box-like	-1970	CTTCTG
E-box	-1891	CATTTG
E-box	-1883	CAATTG
E-box	-1772	CAATTG
E-box	-1744	CAAATG
E-box-like	-1688	CGAATG
E-box-like	-1451	CAGTCG
E-box-like	-1444	CGTGTG
E-box-like	-1325	CGTTTG
E-box-like	-1075	CAAACG
E-box-like	-907	CGTTTG
E-box	-812	CAAATG
E-box-like	-737	CAAACG
E-box-like	-642	CTTATG
E-box-like	-551	CAAAAG
E-box	-539	CAAATG
E-box	-517	CATATG
E-box-like	-493	CCCATG
E-box-like	-461	CATCGG
E-box-like	-397	CATTAG
E-box-like	-301	CACGGG
E-box-like	-253	CAAGGG
E-box-like	-185	CTCATG
E-box-like	-164	CCTATG
E-box-like	-121	CTAATG
E-box	-104	CACCTG
E-box-like	-101	CTGATG

Table S3. List of the primers used in the study.

Gene Name	Primer Sequence
SlbHLH95-OE	F 5'-TCTAGAATGGATTCATCAGAGTTTGAG-3'
	R 5'-GAGCTCTCAATCTGGTTTCTGCATTG-3'
qRT-SlbHLH65	F 5'-CAGAAAGAGCAAGGAGAGAGAAG-3'
	R 5'-GGGACTGTACATGGTTGATGAT-3'
qRT-SlbHLH95	F 5'-TTTGTCACACCACCTAAGTTTACC-3'
	R 5'-CGTGTTCTTCTCACCCTTTCTG-3'
qRT-SlActin	F 5'-TGTCCCTATTTACGAGGGTTATGC-3'
	R 5'-CAGTTAAATCACGACCAGCAAGAT-3'
SlbHLH95-eGFP	F 5'- TCTAGAATGGATTCATCAGAGTTTGAG-3'
	R 5'- GCGGCCGCATCTGGTTTCTGCATTGCTG-3'
qRT-SlWoolly	F 5'-AGGAATTGGGAAGAAGACTTGG-3'
	R 5'-TCATGTTGAGCCTTCATCTGAG-3'

qRT-SlCycB2	F 5'-ATCTAGTGGGTGGTCAGGG-3'<br>R 5'-GCATATTTCCTCGTGATGGGTC-3'
qRT-SlGASA4	F 5'-GCCAAGATTGTTTCAGTTCTCC-3'<br>R 5'-CATACTTCTTAGCATAGTGGTGG-3'
qRT-SlGAMYB2	F 5'-CTAGTGGAAGCTCATCAGAGG-3'<br>R 5'-GGTCCGTAATCAAACTTCTCCTG-3'
qRT-SlANT1	F 5'-TTGAGGTGGCTGAATTATCTAAGG-3'<br>R 5'-CAATAAGTGACCATCTGTTGCC-3'
qRT-SlKS5	F 5'-CATTACAAAGTTGCTCATGACC-3'<br>R 5'-CAATGTGTAGTTCATCTTCCCA-3'
qRT-SlGA20ox2	F 5'-TTTCCATATTCTACCCTACAAG-3'<br>R 5'-TCATCGCATTACAATACTCTT-3'
SlbHLH95-Luciferase	F 5'-TCTAGAATGGATTCATCAGAGTTTGAG-3'<br>R 5'-GGATCCTCAATCTGGTTTCTGCATTG-3'
proSlGA20ox2-Luciferase	F 5'-ACGCGTCGACTTCTTAACAAATCAAAGAAGA-3'<br>R 5'-GGATCCGAGTGGGTAACAAAATATTGGATTC-3'
proSlKS5-Luciferase	F 5'-TCCCCCGGGTTGTGGTATATACAAGCTTTGTA-3'<br>R 5'-CGCGGATCCTATATTGTGGAATATTCAAGGA-3'
SlbHLH95-Y1H	F 5'-GCGCCGTCTCGCTCGAATGGATTCATCAGAGTTTGAGAAAAAC-3'<br>R 5'-GCGCCGTCTCGCTCAAAGCTCAATCTGGTTTCTGCATTGCTGAGC-3'
proSlGA20ox2-Y1H (1×)	5'-CATATATTTTCAAGAGAATATTTTTTTGCTTGTCACAAAATACGTCA<br>GTAGAAAAAAATTGAG-3'
△proSlGA20ox2-Y1H (1×)	5'- CATATATTTTACAGGAAATATTTTTTTGCTTGTCACAAAATACGTA<br>CGTGAAAAAAATTGAG-3'
proSlKS5-Y1H (1×)	5'-ATGACGGTGTCAGACACCTGATGAATTTTAATGACGGTGTCAGACA<br>CCTGATGAATTTTAATGACGGTGTCAGACACCTGATGAATTTT-3'
△proSlKS5-Y1H (1×)	5'-ATGACGGTGTCAGAACCCGTATGAATTTTAATGACGGTGTCAGAAC<br>CCGTATGAATTTTAATGACGGTGTCAGAACCCGTATGAATTTT-3'
